# Genomic context analysis enables the discovery of an unusual NAD‐dependent racemase in phosphonate catabolism

**DOI:** 10.1111/febs.70130

**Published:** 2025-05-19

**Authors:** Francesca Ruffolo, Silvia Conciatori, Giovanni Merici, Tamara Dinhof, Jason P. Chin, Claudio Rivetti, Andrea Secchi, Katharina Pallitsch, Alessio Peracchi

**Affiliations:** ^1^ Department of Chemistry, Life Sciences and Environmental Sustainability University of Parma Italy; ^2^ Institute of Organic Chemistry University of Vienna Austria; ^3^ Vienna Doctoral School in Chemistry (DoSChem), University of Vienna Austria; ^4^ School of Biological Sciences and Institute for Global Food Security Queen's University Belfast UK

**Keywords:** 2‐amino‐1‐hydroxyethylphosphonate, *Alphaproteobacteria*, NAD‐dependent isomerase, phosphonate degradation, racemase

## Abstract

Phosphonates are organic molecules containing a direct carbon–phosphorus (C–P) bond. They are chemically sturdy compounds that can, however, be degraded by environmental microorganisms. In the frame of bacterial phosphonate catabolism, we recently reported the discovery of (*R*)‐1‐hydroxy‐2‐aminoethylphosphonate ammonia‐lyase (PbfA), a lyase acting on the natural compound (*R*)‐2‐amino‐1‐hydroxyethylphosphonate (*R*‐HAEP). PbfA converts *R*‐HAEP into phosphonoacetaldehyde (PAA), which can be subsequently processed and cleaved by further enzymes. However, PbfA is not active toward *S*‐HAEP (the enantiomer of *R*‐HAEP), whose metabolic fate remained unknown. We now describe the identification of a racemase, discovered through genomic context analysis, which converts *S*‐HAEP into *R*‐HAEP, thereby enabling degradation of *S*‐HAEP. We propose for this enzyme the official name 2‐amino‐1‐hydroxyethylphosphonate racemase (shorthand PbfF). To our knowledge, PbfF is the first NAD‐dependent racemase ever described and is structurally unrelated to other known NAD‐dependent isomerases. The enzyme uses NAD^+^ as a cofactor, is inhibited by NADH, and shows catalytic parameters comparable to those of other racemases acting on similar substrates. The presence of a pathway for the breakdown of *S*‐HAEP in numerous bacteria suggests that this compound may be more common in the environment than currently appreciated. Notably, the route for *S*‐HAEP degradation appears to have developed through a mechanism of retrograde metabolic evolution.

## Graphical abstract

The authors identify PbfF (previously annotated as a NAD‐dependent dehydrogenase) as a hitherto unknown enzyme in phosphonate catabolism. Guided by genome context analysis, they show that PbfF is actually a racemase, serving to degrade the natural compound (*S*)‐2‐amino‐1‐hydroxyethylphosphonate (*S*‐HAEP). Conversion of *S*‐HAEP into its enantiomer is a necessary step in its catabolism. PbfF is an efficient enzyme that uses NAD^+^ as a cofactor and is not evolutionarily related to other known isomerases.

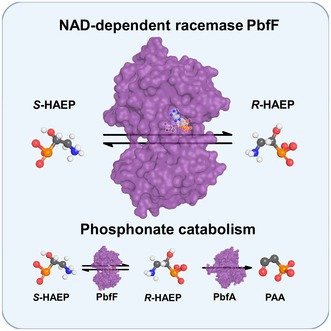

**Abbreviations**
ADH: alcohol dehydrogenase
AEP: 2‐aminoethylphosphonate
Bis‐Tris Propane: 1,3‐bis(tris(hydroxymethyl)methylamino)propane
HAEP: 2‐amino‐1‐hydroxyethylphosphonate
PAA: phosphonoacetaldehyde
PbfA: (*R*)‐2‐amino‐1‐hydroxyethylphosphonate ammonia‐lyase
PbfF: HAEP racemase
PhnA: phosphonoacetate hydrolase
PhnW: AEP:pyruvate aminotransferase
PhnX: PAA hydrolase
PhnY: PAA dehydrogenase
SEC: size‐exclusion chromatography
TEA: triethanolamine

## Introduction

Organophosphonates (herein termed phosphonates for brevity) are organic compounds containing a direct carbon‐phosphorus bond [1]. They regularly occur in the environment, both as natural products [2, 3, 4] and as anthropogenic pollutants [5, 6]. Despite the remarkable stability of the C‐P bond, numerous environmental microorganisms are capable of catabolizing phosphonates–primarily to retrieve phosphorus in nutrient‐limited environments, but also, in many cases, to obtain energy, carbon, and/or nitrogen [7, 8, 9, 10, 11].

**Fig. 1:**
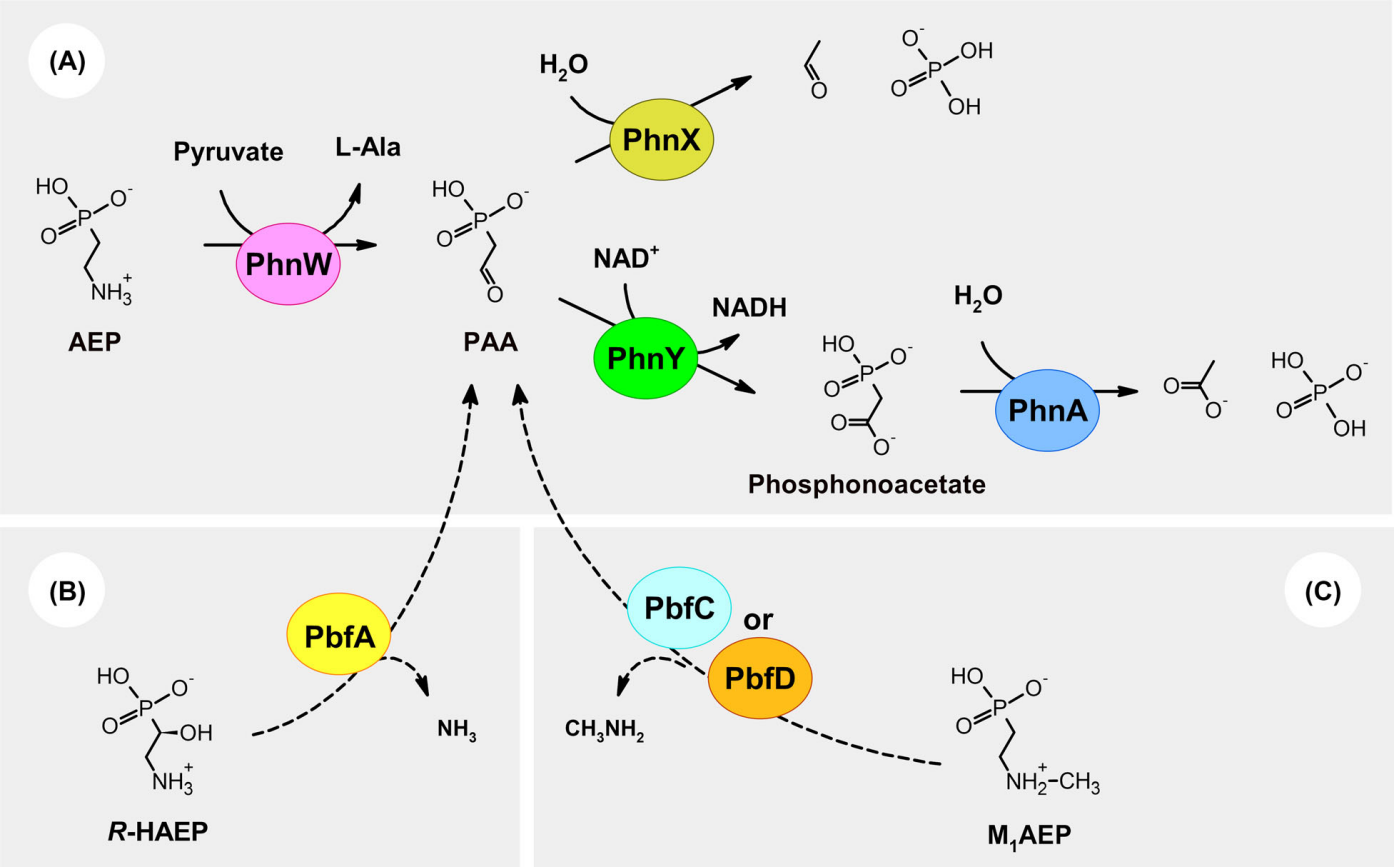
Hydrolytic pathways for the microbial catabolism of 2‐aminoethylphosphonate (AEP) and of some related compounds. (A) For AEP, the first step is the conversion to phosphonoacetaldehyde (PAA), typically operated by the transaminase PhnW (or more rarely by an amine oxidase; not shown [22]). PAA can then be hydrolyzed into acetaldehyde and phosphate by PhnX (upper route; PhnWX pathway), or, alternatively, it can be first converted to phosphonoacetate and then to acetate and phosphate by two consecutive reactions, catalyzed by the enzymes PhnY and PhnA (lower route; PhnWYA pathway). (B) The natural compound *R*‐HAEP can also be converted to PAA through the action of the lyase PbfA [21]. (C) The *N*‐monomethylated form of AEP (M_1_AEP) generates PAA through an oxidative deamination, which can be catalyzed by a FAD‐dependent dehydrogenase (PbfC) or by a FAD‐dependent oxidase (PbfD) [22].

Notably, many bacteria possess so‐called “hydrolytic” pathways for the breakdown of 2‐aminoethylphosphonate (AEP, the most prevalent natural phosphonate [2, 3, 4]). These pathways begin with the conversion of AEP to phosphonoacetaldehyde (PAA), typically carried out by the transaminase PhnW (Fig. 1A) [12, 13, 14]. Thereafter, PAA can be either hydrolyzed into acetaldehyde and phosphate by an enzyme called PhnX (PhnWX pathway) [15, 16, 17] or oxidized by the dehydrogenase PhnY to yield phosphonoacetate, which is then cleaved by the hydrolase PhnA to acetate and phosphate (PhnWYA pathway) (Fig. 1A) [18, 19, 20].

These two pathways are highly specific for AEP, as they cannot effectively process several other natural compounds related to AEP. However, the substrate versatility of the hydrolytic pathways is often expanded by ancillary enzymes that convert other phosphonates into PAA. Indeed, by combining bioinformatics, organic chemistry, and enzymology, our group has identified and characterized two such types of enzymes.

One enzyme, termed PbfA (for “phosphonate breakdown factor A”), is a lyase encoded in over 13% of the bacterial gene clusters dedicated to the hydrolytic pathways [21]. PbfA acts on the natural compound (*R*)‐2‐amino‐1‐hydroxyethylphosphonate (*R*‐HAEP), catalyzing a 1,2‐elimination reaction that directly generates PAA (Fig. 1B) [21].

A second type of recurrent ancillary enzymes consists of FAD‐dependent amine oxidoreductases, subdivisible into at least three distinct structural subgroups (PbfB, PbfC, and PbfD). Representatives of the PbfC and PbfD subgroups were characterized in detail, revealing that—in spite of significant mechanistic differences—these enzymes preferentially oxidize another common natural phosphonate, *N*‐methyl AEP (M_1_AEP), which cannot be processed by PhnW. Again, the reactions catalyzed by these enzymes yield PAA (Fig. 1C) [22]. There is evidence that PbfB enzymes also catalyze this reaction, although the mechanistic details remain elusive [22].

The present study deals with a new ancillary enzyme, termed PbfF. This was annotated as a NAD(P)‐dependent dehydrogenase, and genome context suggested a link between its function and the function of PbfA. We demonstrate here that PbfF is actually a NAD‐dependent racemase, capable of producing *R*‐HAEP from its enantiomer, *S*‐HAEP. Since *S*‐HAEP is not a substrate for PbfA, its isomerization is a necessary step toward degradation. PbfF apparently occurs only in some species of *Alphaproteobacteria* and is structurally very distinct from other NAD‐dependent enzymes that catalyze analogous isomerization reactions.

## Results

### Identification of PbfF—a putative dehydrogenase encoded in AEP‐degradation gene clusters from *Alphaproteobacteria*

Our work leverages the well‐known phenomenon whereby bacterial genes involved in the same cellular function are commonly grouped in clusters or operons [23, 24]. Accordingly, we performed a systematic examination of the bacterial gene clusters dedicated to the hydrolytic pathways for AEP catabolism. We searched in particular for genes of unknown function that occurred with some frequency in these clusters. Our assumption was that such genes might encode ancillary enzymes able to feed the AEP catabolic pathways using different AEP‐related compound(s).

During this inspection, we noticed that several AEP degradation clusters in *Alphaproteobacteria* comprise a gene encoding a putative NAD‐dependent dehydrogenase (pfam02826). The presence of this gene did not seem accidental, as it occurred across various species within the *Hyphomicrobiales* and *Rhodobacterales* orders. Furthermore, although the putative dehydrogenase was predominantly found in clusters of the *phnWYA* type, in at least one genome (*Acidimangrovimonas sediminis*) it was included in a *phnWX*‐type cluster. These observations strongly supported a role of the putative dehydrogenase gene in phosphonate catabolism. Accordingly, we termed the encoded protein “phosphonate breakdown factor F” (PbfF).

**Fig. 2:**
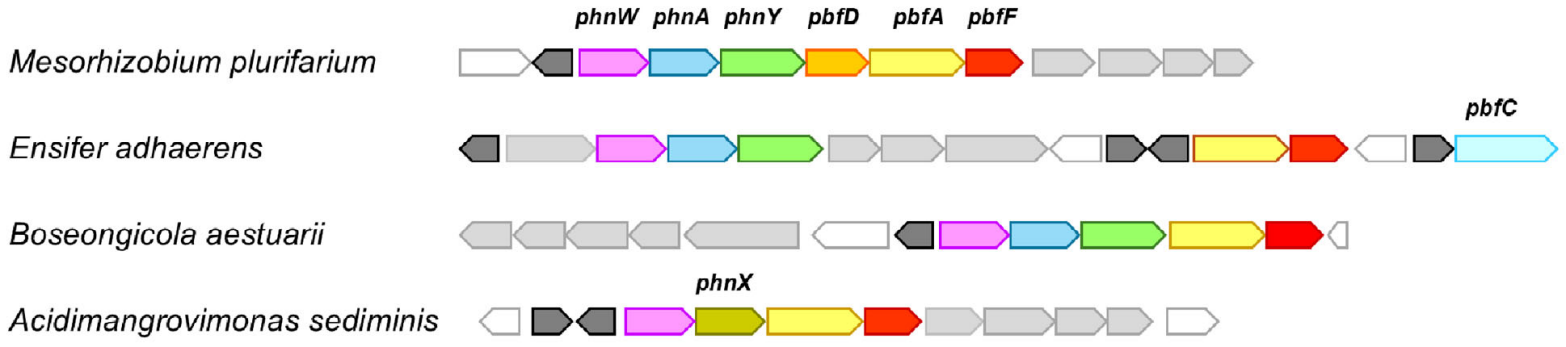
Genes coding for a predicted NAD‐dependent dehydrogenase (PbfF) in clusters dedicated to AEP degradation. The *pbfF* genes are shown in red; the accession IDs of the encoded proteins are provided in Table 1. Other highlighted genes include *phnW* (light purple), *phnA* (azure), *phnY* (green), *phnX* (olive), *pbfA* (yellow), *pbfC* (ice blue) and *pbfD* (orange). Putative transporter genes are shown in light gray, whereas predicted transcription regulators are in dark gray.

Examples of clusters encompassing the *pbfF* gene are depicted in Fig. 2, whereas a more extensive list of organisms' names and sequence accession IDs is provided in Table 1.

**Table 1:** A representative list of *Alphaproteobacteria* whose AEP‐degradation clusters contain the *pbfF* gene.

Organism	Order	NCBI accession ID	Cluster type	Other genes in cluster
*Mesorhizobium plurifarium*	*Hyphomicrobiales*	CDX32929	*phnWYA*	*pbfA, pbfD*
*Mesorhizobium opportunistum*	*Hyphomicrobiales*	WP_013896951	*phnWYA*	*pbfA, pbfC*
*Mesorhizobium ciceri*	*Hyphomicrobiales*	WP_013533036	*phnWYA*	*pbfA, pbfC*
*Labrys sp. WJW*	*Hyphomicrobiales*	WP_245314481	*phnWYA*	*pbfA, pbfD*
*Ensifer adhaerens*	*Hyphomicrobiales*	WP_209789860	*phnWYA*	*pbfA. pbfC*
*Bosea lupini*	*Hyphomicrobiales*	WP_244543933	*phnWYA*	*pbfA. pbfC*
*Bosea robiniae*	*Hyphomicrobiales*	WP_310090909	*phnWYA*	*pbfA. pbfC*
*Hoeflea sp. IMCC20628*	*Hyphomicrobiales*	WP_047030208.	*phnWYA*	*pbfA. pbfC*
*Shinella zoogloeoides*	*Hyphomicrobiales*	WP_119255852	*phnWYA*	*pbfA. pbfC*
*Roseovarius faecimaris MME‐070*	*Rhodobacterales*	WP_157708322	*phnWYA*	*pbfA*
*Roseovarius (Pelagivirga) litorisediminis*	*Rhodobacterales*	WP_085890473	*phnWYA*	*pbfA, pbfC*
*Roseovarius nanhaiticus*	*Rhodobacterales*	WP_076535357	*phnWYA*	*pbfA, pbfC*
*Aliiroseovarius crassostreae*	*Rhodobacterales*	WP_055187564	*phnWYA*	*pbfA*
*Boseongicola aestuarii*	*Rhodobacterales*	WP_093973731	*phnWYA*	*pbfA*
*Shimia aestuarii*	*Rhodobacterales*	WP_093096494	*phnWYA*	*pbfA*
*Rhodovulum euryhalinum*	*Rhodobacterales*	WP_132540488	*phnWYA*	*pbfA. pbfC*
*Acidimangrovimonas (Defluviimonas) sediminis*	*Rhodobacterales*	WP_102222898	*phnWX*	*pbfA*

The pfam02826 family, to which PbfF belongs, is annotated as “d‐isomer specific 2‐hydroxyacid dehydrogenase family”. Indeed, PbfF resembled most closely enzymes such as d‐3‐phosphoglycerate dehydrogenase, d‐glycerate dehydrogenase (also known as hydroxypyruvate reductase), or d‐lactate dehydrogenase, even though the identity to functionally validated enzymes was in all cases <36% (Table 2).

**Table 2:** Sequence comparison between PbfF from *Mesorhizobium plurifarium* and the most similar enzymes of known function (identified by blasting the UniProt KB/Swiss‐Prot database). Percent identities were calculated as described in the Methods.

Enzyme	Cofactor	Organism	UniProt ID	% identity	Ref.
d‐3‐phosphoglycerate dehydrogenase	NAD	*Mycobacterium tuberculosis*	P9WNX3	35.7%	[25]
d‐3‐phosphoglycerate dehydrogenase	NAD	*Homo sapiens*	O43175	34.8%	[26]
d‐glycerate dehydrogenase	NAD	*Methylorubrum extorquens*	Q59516	35.4%	[27]
Hydroxypyruvate reductase	NADP	*Escherichia coli*	P37666	30.0%	[28]
d‐lactate dehydrogenase	NAD	*Thermodesulfatator indicus*	F8A9V0	33.4%	[29]
d‐lactate dehydrogenase	NAD	*Pseudomonas aeruginosa*	Q9I530	31.9%	[30]

### Hypotheses on the function of PbfF

As shown in Table 1, the clusters containing *pbfF* invariably also included *pbfA*, which encodes the lyase that degrades *R*‐HAEP (Fig. 1B). This co‐occurrence seemed highly significant, as the *pbfA* gene, *per se*, is found in <25% of all hydrolytic clusters [21]. Furthermore, in most cases, the *pbfA* and *pbfF* genes were not just co‐present but also physically adjacent (Fig. 2). These observations suggested a connection between the function of PbfF and the catabolism of *R*‐HAEP.

**Fig. 3:**
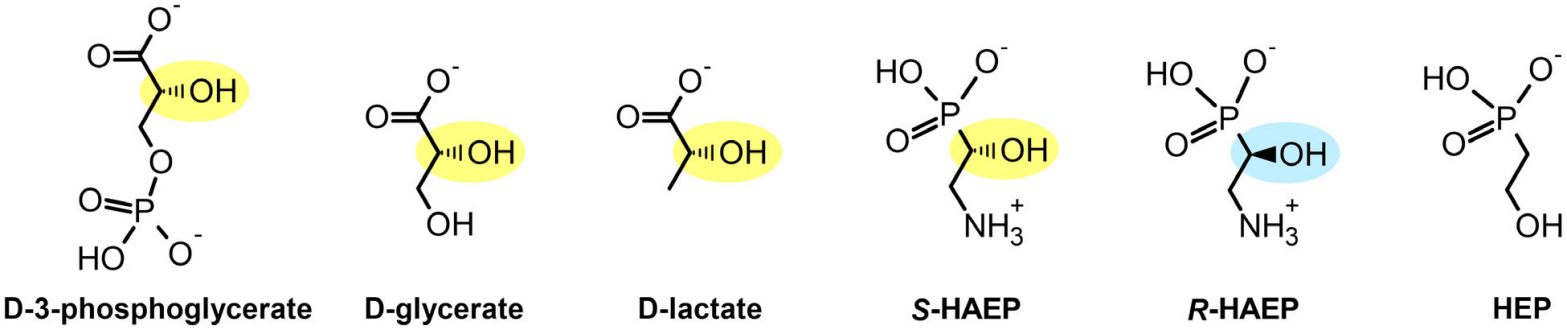
Structural comparison between the substrates of d‐hydroxyacid dehydrogenases and potential substrates of PbfF. The d‐hydroxyacids are shown on the left and some naturally occurring hydroxy‐containing phosphonates are shown on the right. Chiral centers, when present, are highlighted in yellow or light blue.

A second aspect worth noting is that all the dehydrogenases homologous to PbfF act on a chiral alcohol group whose stereochemistry is opposite to that of *R*‐HAEP (Fig. 3). This suggested that PbfF could be involved in the degradation of the *S*‐enantiomer of HAEP (that is, *S*‐HAEP), which was shown to occur in nature, albeit in a very narrow context [31]. Based on the points above, we formulated three hypotheses about the function of PbfF.

In a first scenario, PbfF could act similarly to d‐lactate dehydrogenase and d‐glycerate dehydrogenase, oxidizing *S*‐HAEP to form the corresponding α‐oxophosphonate (2‐amino‐1‐oxo AEP; OAEP) and NAD(P)H. To our knowledge, OAEP has never been found in nature. However, if it was formed by PbfF, the structural similarity to *R*‐HAEP might favor its processing by PbfA, possibly together with PhnW, to eventually yield PAA.

Alternatively, PbfF could act as a racemase rather than as a dehydrogenase, isomerizing *S*‐HAEP to *R*‐HAEP via an oxidation (to transiently form OAEP) followed by a reduction on the opposite face of the carbonyl group. A similar mechanism has been described previously for a handful of NAD‐dependent isomerases, the best studied being UDP‐galactose 4‐epimerase [32, 33]. A racemase role for PbfF would explain very straightforwardly the connection between PbfF and PbfA; however, no known isomerases are classified within the pfam02826 structural family.

A third, albeit less likely possibility was that PbfF could serve to oxidize 2‐hydroxyethylphosphonate (HEP), another naturally occurring compound [34]. The oxidation of HEP would directly generate PAA, feeding seamlessly into the hydrolytic pathways. However, as compared to *S*‐HAEP, HEP exhibits less structural similarity to the substrates of pfam02826 enzymes (Fig. 3). Moreover, a HEP dehydrogenase function would not account for the tight genomic connection between PbfF and PbfA.

### PbfF allows the degradation of *S*‐HAEP

To experimentally address the catalytic function of PbfF, we obtained a synthetic gene encoding the enzyme from *Mesorhizobium plurifarium*, cloned into an expression vector. The gene was expressed in *E. coli*, and the recombinant His‐tagged protein was purified by Ni‐affinity chromatography, as detailed in the Methods. The purified PbfF contained <0.8% bound NAD(P)H or NAD(P)^+^ (based on UV–VIS absorption; see Methods) and when subjected to size‐exclusion chromatography, it displayed an apparent size of ~67 kDa, suggesting that the protein exists in solution as a dimer (not shown; the calculated monomeric mass of PbfF is 37.2 kDa).

A first functional test was conducted to establish whether PbfF helps degrade different potential substrates. The test relied on the detection of inorganic phosphate; the rationale was that if PbfF was capable of degrading *S*‐HAEP or HEP, either alone or in combination with PbfA and/or PhnX, this would lead to the accumulation of phosphate, which could then be conveniently detected by the BIOMOL Green^®^ assay.

**Fig. 4:**
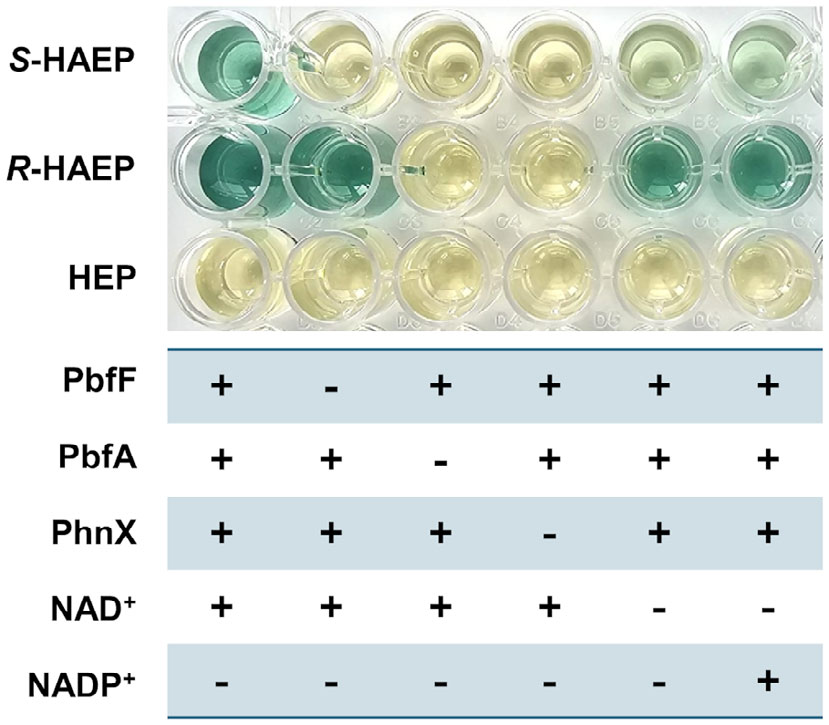
A phosphate release assay shows the involvement of PbfF in the degradation of *S*‐HAEP. Different phosphonates (*S*‐HAEP, *R*‐HAEP or HEP, 3 mm) were tested as described in the Methods. PbfF, in combination with PbfA and PhnX, afforded phosphate release from *S*‐HAEP. The presence of both PbfF and PbfA was essential: as much as PbfA alone is unable to process *S*‐HAEP [21], PbfF, in the absence of PbfA, could not accomplish the degradation of *S*‐HAEP (nor of *R*‐HAEP). The experiment was replicated three times with essentially identical results.

The results of this experiment, conducted in microtiter format, were revealing (Fig. 4). Phosphate was not released from HEP when this compound was incubated in the presence of PbfF plus PhnX, as it would be expected if PbfF was oxidizing HEP to PAA. The same was true even in the presence of PbfA and irrespective of the presence or absence of exogenous NAD^+^ or NADP^+^. These results excluded that PbfF could act as a HEP dehydrogenase or anyhow be involved in the degradation of HEP.

In contrast, *S*‐HAEP was evidently degraded (yielding inorganic phosphate) when incubated with PbfF, PbfA, and PhnX. Omission of any of the proteins resulted in no detectable phosphate release, suggesting that the three enzymes were working as a pathway. Furthermore, degradation of *S*‐HAEP was robust only when the reaction mixture was supplemented with NAD^+^; only a very weak signal was detected in the presence of NADP^+^ or in the absence of added coenzymes (presumably due to traces of NAD^+^ carried over from the protein purification procedure) (Fig. 4). Since PhnW was not present in any of the assays of Fig. 4, its activity was not required for *S*‐HAEP degradation.

### Evidence that PbfF is a HAEP racemase

While the results above did not rigorously exclude that PbfF could act as a *S*‐HAEP dehydrogenase, they were most easily explained by assuming that PbfF converts *S*‐HAEP into *R*‐HAEP. The following biochemical evidence further supports the notion that PbfF is indeed a racemase.

**Fig. 5:**
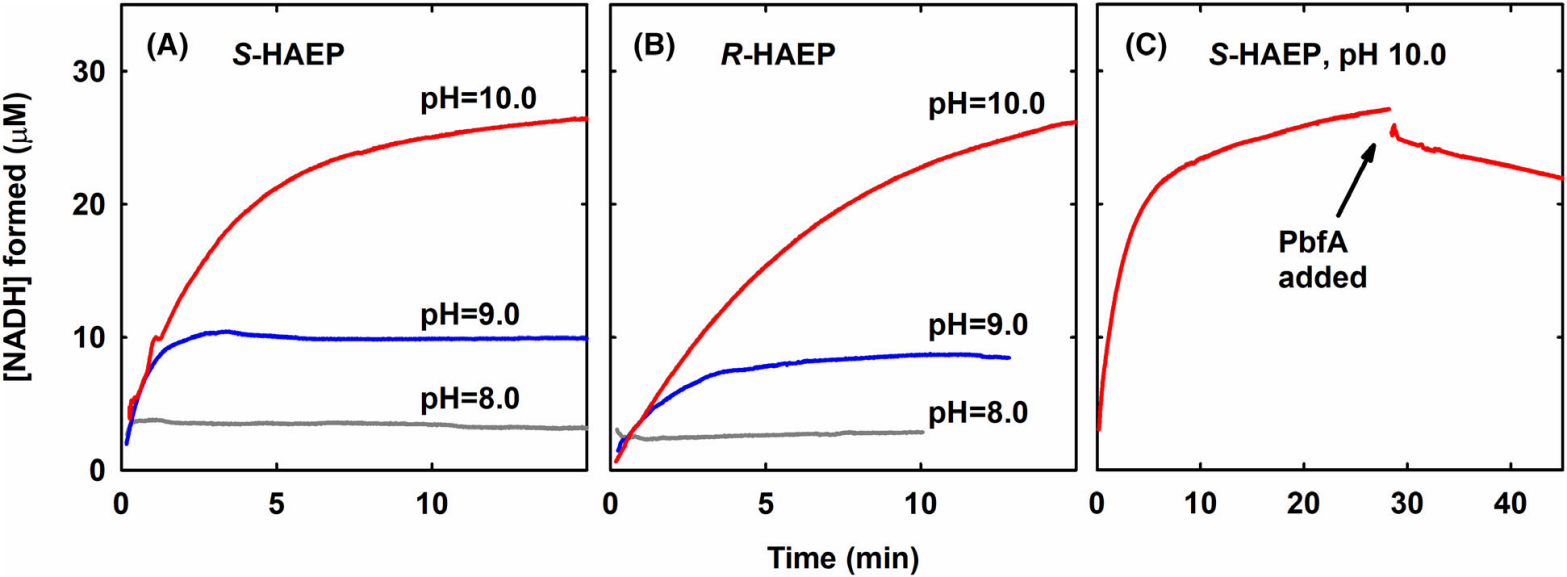
Accumulation of NADH upon incubation of PbfF with HAEP isomers. Each kinetic trace is representative of at least three repetitions. (A) Reaction of PbfF with *S*‐HAEP. The enzyme (0.9 μm) to a solution containing 3 mm *S*‐HAEP and 0.3 mm NAD^+^ in 50 mm Bis‐Tris propane at three different pH values, 23 °C. The amount of NADH formed was calculated based on the increase of absorbance at 340 nm. At pH 8 NADH formation was almost undetectable. Even at pH 10 (where NAD^+^ reduction is intrinsically favored) the NADH accumulated in 15 min was <10% of the total NAD. (B) Reaction of PbfF with *R*‐HAEP. Apart from the presence of 3 mm *R*‐HAEP (in place of *S*‐HAEP) other conditions were as in panel A. (C) Effect of the addition of PbfA on the accumulation of NADH upon reaction of PbfF with *S*‐HAEP. The reaction was conducted as in panel A (pH 10.0) except that after about 28 min the reaction mixture was supplemented with 2 μm PbfA.

First, if PbfF was an *S*‐HAEP dehydrogenase, NADH would be one of the products. In contrast, for a complete racemase reaction, NADH would be just an intermediate. Indeed, when PbfF was incubated with *S*‐HAEP and NAD^+^, the formation of NADH was very modest – even when a large concentration of NAD^+^ was present and even at high pH, where NADH formation is inherently favored (Fig. 5A).

Second, if PbfF was a *S*‐HAEP dehydrogenase, it would be presumably stereospecific and unable to react with *R*‐HAEP. In contrast, a racemase should, by definition, react with both *S*‐HAEP and *R*‐HAEP. Indeed, incubation of PbfF with *R*‐HAEP and NAD^+^ led to the formation of NADH, very much as observed with *S*‐HAEP (Fig. 5B).

Third, if PbfF was a dehydrogenase producing OAEP and NADH, and if PbfA were somehow consuming OAEP, the presence of PbfA in the reaction mixture should drive the PbfF reaction, favoring the accumulation of NADH. However, the opposite was observed, as it might be expected in case PbfF is a racemase (Fig. 5C).

**Fig. 6:**
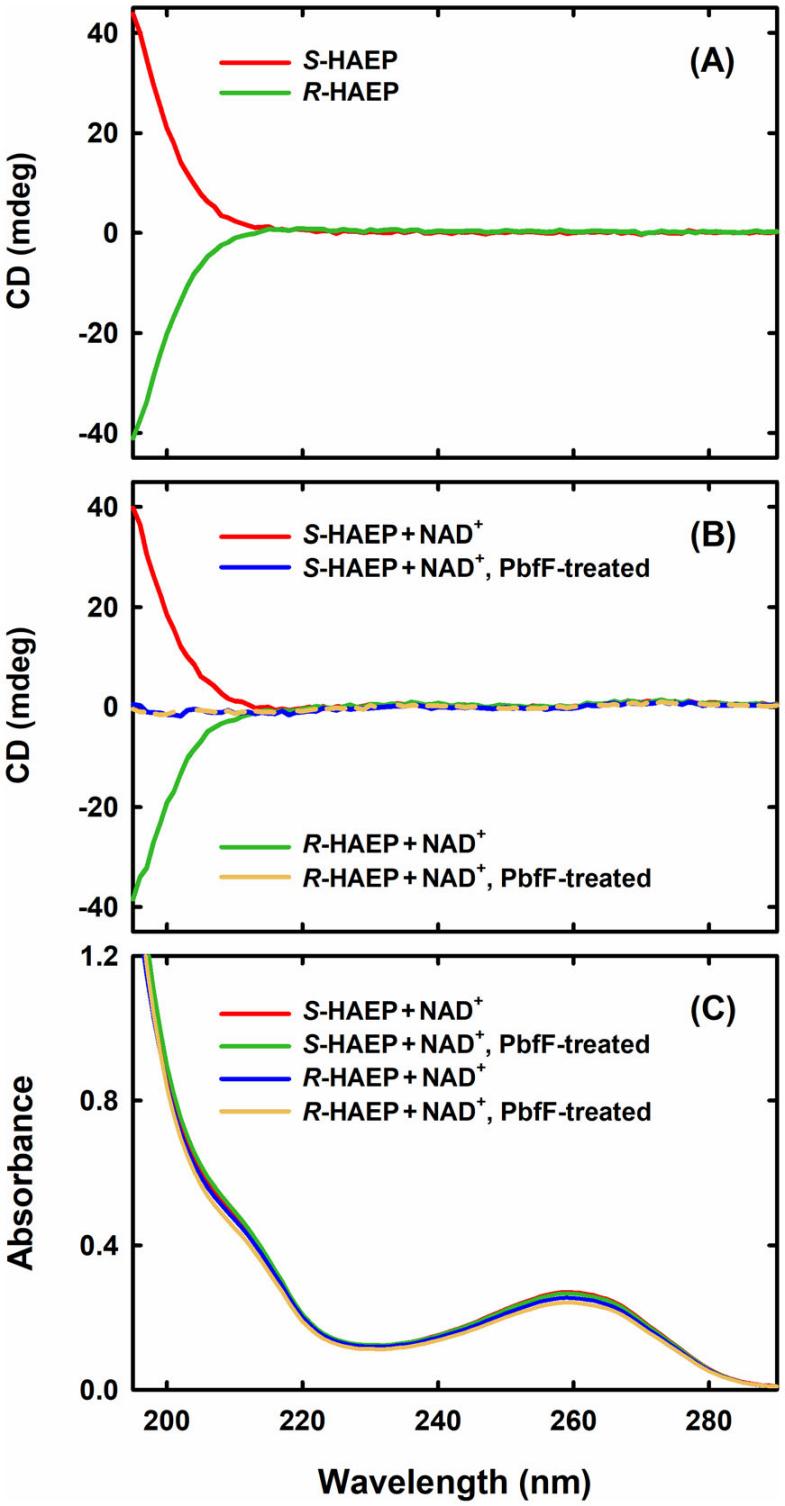
Circular dichroism (CD) assessment of the racemase activity of PbfF. Each reported experiment is representative of three repetitions. (A) CD spectra of *S*‐HAEP and *R*‐HAEP. Solutions of the two enantiomers (5 mm in each case, pH 8.0) were placed in a 1 mL quartz cuvette with 0.5 cm path length and spectra were collected using a J‐1500 CD spectrophotometer (Jasco Inc., Easton, MD, USA). Bandwidth 1 nm, scanning speed 50 nm·min^−1^. (B) CD signals of the HAEP enantiomers disappear upon PbfF treatment. Solutions initially containing *S*‐HAEP (5 mm, pH 8.0) and NAD^+^ (50 μm) were incubated for 75 min in the absence (red line) or in the presence (blue line) of 1 μm PbfF. Solutions with *R*‐HAEP and NAD^+^ were also incubated for 75 min in the absence (green line) or in the presence (yellow line) of 1 μm PbfF. All solutions were ultrafiltered before collecting the spectra. (C) UV absorption spectra of the same samples as in panel B show no disappearance of chromophoric species due to PbfF. The band at ~260 nm and the shoulder at ~215 nm are attributable to NAD^+^.

Ultimately, the ability of PbfF to invert the absolute configuration of the HAEP chiral center was assessed by CD spectroscopy. In fact, HAEP shows a weak UV absorption below 210 nm. At these low wavelengths, *S*‐HAEP shows a positive CD signal, whereas *R*‐HAEP shows a negative signal (Fig. 6A). Treatment of either *S*‐HAEP or *R*‐HAEP with PbfF (in the presence of NAD^+^) led to the disappearance of the CD signal, as expected for the conversion of either starting HAEP isomer into a racemic mixture (Fig. 6B). The disappearance of the CD signal did not correspond to significant changes in the absorption spectrum, again as expected for a racemization reaction (Fig. 6C).

### Initial kinetic characterization of the PbfF reaction

To conveniently monitor the PbfF activity, we devised a continuous spectrophotometric assay in which the racemase reaction was coupled to three consecutive reactions catalyzed by PbfA, PhnX, and by a bacterial alcohol dehydrogenase (EC 1.1.1.2). The latter enzyme would use NADPH to reduce the acetaldehyde generated by PhnX, leading to a decrease in NADPH absorbance at 340 nm. The choice of an NADP‐dependent dehydrogenase was meant to limit any possible interference of the coupling enzyme with the activity of PbfF, which requires NAD^+^ and reacts poorly with NADP^+^ (Fig. 4). Furthermore, the coupled assay was conducted at pH 8, where the conversion of NAD^+^ to NADH by PbfF is negligible (Fig. 5A) and hence does not affect the readout at 340 nm.

**Fig. 7:**
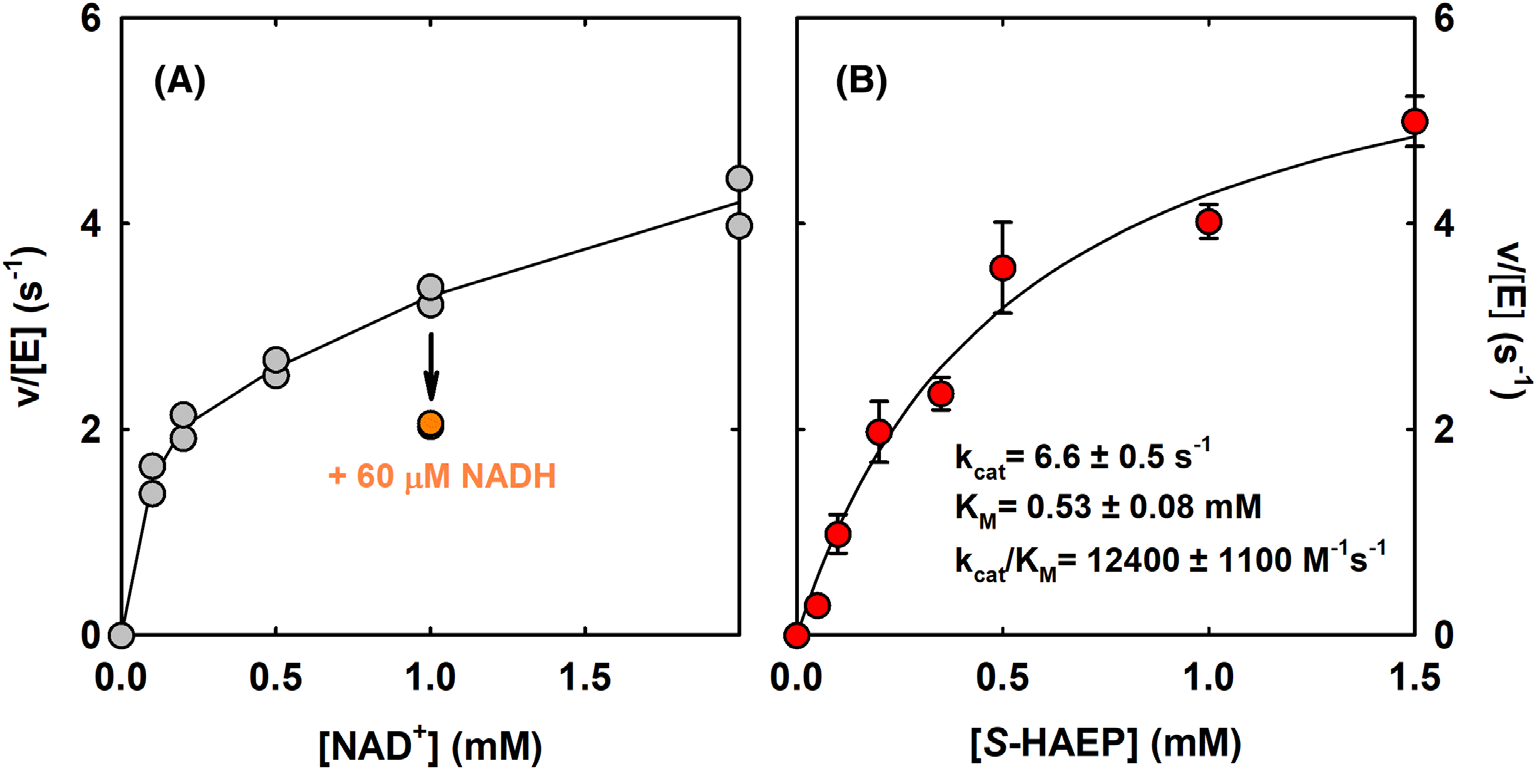
PbfF activity as a function of the concentrations of cofactor and of substrate (50 mm TEA‐HCl, pH 8.0, 25 °C). (A) Dependence of PbfF activity on exogenous NAD^+^. In addition to PbfF (35 nm) the reaction mixture contained 1 mm *S*‐HAEP, 5 mm MgCl_2_, 7.7 μm PbfA, 3.6 μm PhnX, 6.66 U·mL^−1^ ADH, and 0.3 mm NADPH. For each NAD^+^ concentration tested, two technical replicates were collected (gray circles). The orange circles show the activity in the presence of 1 mm NAD^+^ and 60 mm NADH. (B) Dependence of activity on the concentration of *S*‐HAEP. Conditions were as in panel A, except that NAD^+^ was kept at 1 mm while the concentration of S‐HAEP varied from 0.05 to 1.5 mm. Data points represent the average (±SD) of three technical replicates.

We first assessed the dependence of the PbfF activity on the concentration of exogenous NAD^+^, showing that the enzyme is half‐saturated at ~200 μm of the cofactor (Fig. 7A). In contrast, PbfF was substantially inhibited by NADH: in the presence of 1 mm NAD^+^, its activity was halved when 60 μm NADH was also present in the reaction mixture (Fig. 7A).

We then established the basic catalytic parameters for the racemase reaction. At pH 8.0 (25 °C), PbfF showed *k*_cat_ = 6.6 s^−1^, *K*_M_ = 0.53 mm, and *k*_cat_/*K*_M_ = 12 400 m ^−1^·s^−1^ (Fig. 7B). The catalytic efficiency of PbfF was comparable to the efficiency of other NAD‐dependent isomerases. For example, UDP‐galactose 4‐epimerase from *Aeromonas hydrophila* reportedly shows *K*_M_ = 0.54 mm and *k*_cat_/*K*_M_ = 25 800 m ^−1^·s^−1^ [35]; for UDP‐xylose 4‐epimerase from *Sinorhizobium meliloti* the catalytic parameters were *K*_M_ = 0.31 mm and *k*_cat_/*K*_M_ = 42 100 m ^−1^·s^−1^ [36]. Among NAD‐independent α‐hydroxyacid racemases, lactate racemase from *Lactobacillus plantarum* isomerizes d‐lactate with *K*_M_ = 11 mm and *k*_cat_/*K*_M_ = 121 000 m ^−1^·s^−1^ [37] whereas its homolog from *Isosphaera pallida* shows *K*_M_ = 0.56 mm and *k*_cat_/*K*_M_ = 14 400 m ^−1^·s^−1^ [38].

### AlphaFold 3 prediction of the PbfF three‐dimensional structure and NAD ^+^ binding site

**Fig. 8:**
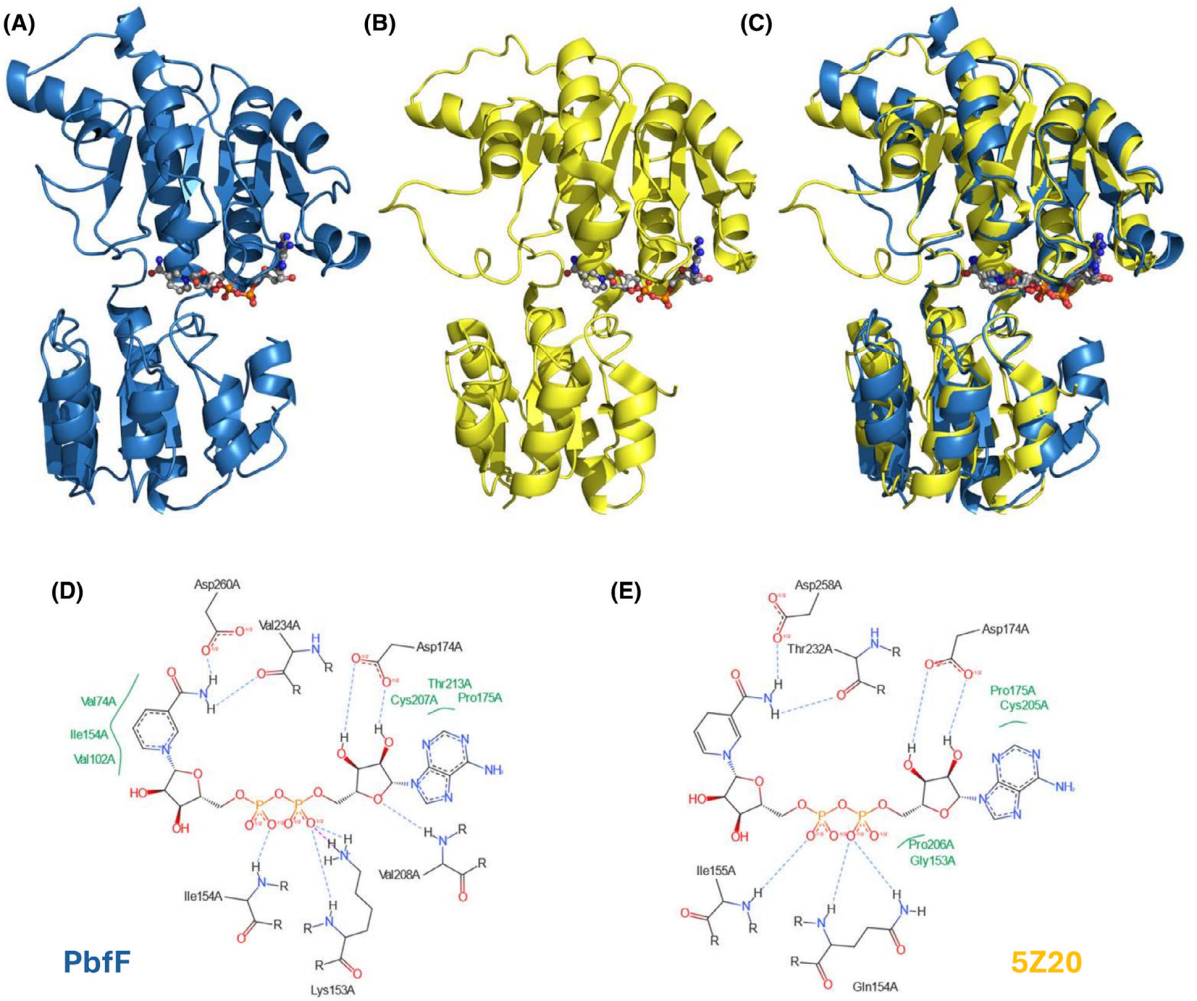
Predicted structure of PbfF from *M. plurifarium* and comparison with the experimental crystal structure of a pfam02826 dehydrogenase. (A) Three‐dimensional structure of the PbfF monomer predicted by AlphaFold 3 [39]. The bound NAD^+^ cofactor is shown in ball‐and‐stick. (B) Structure of d‐lactate dehydrogenase from *P. aeruginosa* (PDB: 5Z20) [30]. (C) Structural alignment of the two structures in panels A and B, generated by PyMOL [41]. (D) Schematic representation of the interactions formed by NAD^+^ bound to the predicted PbfF structure. The interactions were visualized through the PoseEdit [42] web tool (https://proteins.plus). (E) Interactions formed by NADH in the active site of *P. aeruginosa* d‐lactate dehydrogenase. Several of the interactions match closely the predicted interactions at the PbfF active site (panel D). Some of the differences (e.g., the lack of an ionic interaction with the phosphate groups in 5Z20) might be related to the different functions of the two proteins (dehydrogenase vs. isomerase).

We used AlphaFold 3 [39] to predict the structure of PbfF with bound NAD^+^. The best predicted model of the PbfF monomer (iptM = 0.96; pTM 0.91) is shown in Fig. 8A. The three‐dimensional model appeared very consistent with the crystal structures of other pfam02826 enzymes, such as d‐lactate dehydrogenase from *Pseudomonas aeruginosa* [30], shown in Fig. 8B. A structural alignment of the two proteins yielded an RMSD value (referred to α‐carbons) of 0.919 Å, indicating high similarity (Fig. 8C; note that the comparison was conducted at the monomer level; d‐lactate dehydrogenase is a homotetramer [30], whereas PbfF, according to SEC analysis, is a dimer).

In the predicted PbfF structure, NAD^+^ appeared to be held in place by multiple hydrogen bonds, as well as by hydrophobic and ionic interactions (Fig. 8D). The reliability of the predicted NAD^+^ binding mode was supported by two observations. First, the cofactor was placed entirely within the single binding pocket that P2Rank [40] identified in the PbfF structure (see Methods). Furthermore, many (albeit not all) of the predicted interactions between NAD^+^ and PbfF residues matched interactions formed by NADH in the crystal structure of *P. aeruginosa* d‐lactate dehydrogenase (Fig. 8E).

## Discussion

The current, ever‐increasing flood of complete genomic sequences offers both great opportunities and great challenges to researchers. For biochemists and enzymologists in particular, a major endeavor is mining genomic information to identify new enzymes and novel metabolic pathways [43, 44]. In this perspective, while there are many pre‐experimental approaches that can be used to decrypt the function of uncharacterized enzyme‐coding genes, one of the most reliable and informative is arguably the examination of genome context, specifically when dealing with bacterial genomes [45, 46].

Herein, an analysis based on genome context led us to identify a new probable enzyme in phosphonate metabolism, which we termed PbfF. We demonstrated that this enzyme is an isomerase capable of interconverting *S*‐HAEP and *R*‐HAEP, which are both naturally occurring phosphonates, but only the latter can be degraded to PAA by the lyase PbfA. While in principle the role of PbfF could be amphibolic, the tight and constant genomic association of the *pbfF* and *pbfA* genes implies that the biological purpose of PbfF is to assist the degradation of *S*‐HAEP, rather than its biosynthesis.

### A novel NAD‐dependent racemase

To date, there are a few other known NAD‐dependent isomerases that, like PbfF, catalyze the inversion of stereochemistry of chiral secondary alcohols. The most studied of these enzymes is arguably UDP‐galactose 4‐epimerase (GalE) [33, 47]. Other members include UDP‐*N*‐acetylglucosamine 4‐epimerase [48] and UDP‐xylose 4‐epimerase [36]. However, PbfF differs strikingly from these other enzymes for at least two key reasons.

First, GalE and the related isomerases act on a nucleotide‐bound sugar or sugar derivative and operate specifically on one of the different stereocenters of the sugar (whence their classification as epimerases). PbfF, on the other hand, acts on a much smaller substrate that is not bound to a nucleotide and inverts the stereochemistry of its single chiral carbon, formally qualifying as a racemase. We therefore propose for this enzyme the official name of 2‐amino‐1‐hydroxyethylphosphonate racemase.

More relevantly, the PbfF structure is completely different from those of UDP‐galactose 4‐epimerase and the related enzymes, which belong to a distinct structural family (pfam16363) and show percent identities <15% to PbfF. Thus, PbfF and GalE do not appear to be phylogenetically related, implying that their functional analogies are the result of convergent evolution.

What did PbfF evolve from? As noted in the results, the closest PbfF homologs of known function include d‐lactate dehydrogenase (Fig. 8B) and other dehydrogenases acting on similar substrates (Table 2). Therefore, it is likely that PbfF derived from an ancestral d‐α‐hydroxyacid dehydrogenase. Notably, PbfF did *not* derive from known enzymes that racemize small α‐hydroxy acids like lactate and glycerate. In fact, the α‐hydroxy acid racemases identified so far possess a different fold and use a nickel‐containing cofactor instead of NAD^+^ [38].

### On the mechanism of PbfF

Despite the structural differences, the PbfF reaction has obvious similarities with the reactions carried out by GalE and the related epimerases. It seems, hence, instructive to compare the known mechanistic features of GalE with the properties of PbfF as they emerge from the present study.

The known NAD‐dependent epimerases are enzymes that use NAD^+^ not as a substrate (or product) but rather as a stably bound cofactor, which is transiently reduced during catalysis and regenerated at the end of each turnover [33, 47]. For PbfF, NADH also seems to be retained by the protein. In particular, at pH 8.0, the amount of NADH accumulating during catalysis is very small and comparable to the protein concentration (Fig. 5A). Nonetheless, at pH 9.0 and pH 10.0, NADH slowly builds up in substantial molar excess with respect to the enzyme (Fig. 5A), implying that some leaking of NADH occurs over time. This observation, together with the observation that purified PbfF is essentially devoid of bound cofactor (and only barely active in the absence of added NAD^+^), suggests that, mechanistically, PbfF resembles more the mammalian GalE, to which NAD^+^ binds somewhat loosely [49, 50] rather than the *E. coli* enzyme, where the cofactor is very tightly bound [51].

In GalE‐type epimerases, NADH is formed transiently from the oxidation of a chiral alcohol, generating a ketone intermediate that is retained in the enzyme active site, where it can be reduced by NADH on the opposite face of the carbonyl group to yield a stereocenter with inverted configuration [33]. For PbfF, the formation of a nonchiral oxo intermediate (OAEP) is also mechanistically plausible, even though we have no direct evidence for it. We speculate that retaining OAEP in the active site may serve two purposes. First, it would increase catalytic efficiency by avoiding unnecessary dissociation and rebinding steps. Second, it would prevent OAEP from reacting with itself (dimerization) and with other molecules in the cytoplasm. Indeed, aminoketones like OAEP are notoriously reactive and their release may represent a liability for the cell [52].

**Fig. 9:**
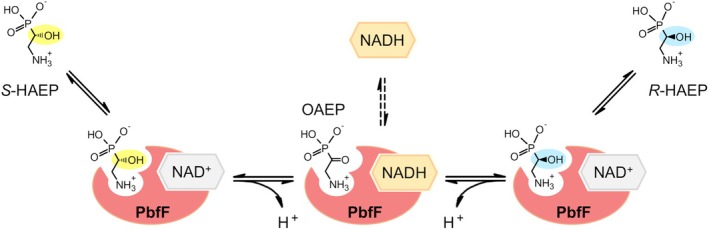
Tentative scheme for the reaction catalyzed by PbfF. The conversion of *S*‐HAEP into *R*‐HAEP proceeds without net production of NADH. However, NADH is formed transiently as an enzyme‐bound intermediate, which is expectedly more stable at high pH (NAD^+^ reduction is accompanied by the release of one proton). Over time, some NADH may also leak out of the active site (dashed vertical arrows).

A tentative kinetic model for the PbfF reaction, based on the experimental data presented in this work and on the analogies with the GalE reaction, is shown in Fig. 9. The model tries to explain not just the *S*‐HAEP to *R*‐HAEP interconversion, but also the modest accumulation of NADH detected in Fig. 5. It posits that the formation of NADH in the enzyme active site is stabilized at high pH and that the reduced cofactor may be slowly released into the medium.

### On the environmental abundance of *S*‐HAEP

The occurrence of an enzyme apparently dedicated to degrading *S*‐HAEP elicits considerations about the distribution and relevance of this compound in the environment. Although information on the natural occurrence of *S*‐HAEP is very limited, it is known that *S*‐HAEP is an intermediate in a pathway for the biosynthesis of the natural product hydroxyphosphonocystoximic acid in *Streptomyces regensis* [31]. Additionally, it was reported that the predatory bacterium *Bdellovibrio stolpii* incorporates HAEP in its membranes, but the enantiomeric form of the phosphonate was not established [53]. The production of HAEP (stereochemistry unspecified) was also postulated for other bacteria, based on genomic observations [54, 55].

PbfF is present in several organisms, suggesting it provides some general biological advantage. *S*‐HAEP could perhaps be a relatively abundant phosphonate in certain environments, making it a valuable food source. It is notable that, in contrast to the *Hyphomicrobiales* spp. (which inhabit a wide range of niches including soil, plant roots, and animals [56]), bacteria belonging to the *Rhodobacterales* order are mostly found in marine environments [57]. This distribution is consistent with the notion that phosphonates are an important source of phosphorus, nitrogen, and carbon in the oceans [58]. However, while compounds such as AEP are known to be abundant in the oceans, *S*‐HAEP has yet to be detected. At any rate, the *Rhodobacterales* and *Hyphomicrobiales* are closely related [59], suggesting vertical inheritance of *pbfF*, although the sparse distribution of the gene within these orders implies that the fitness benefits provided by the consumption of *S*‐HAEP are niche‐specific.

### The catabolism of *S*‐HAEP provides an example of retrograde metabolic evolution

**Fig. 10:**
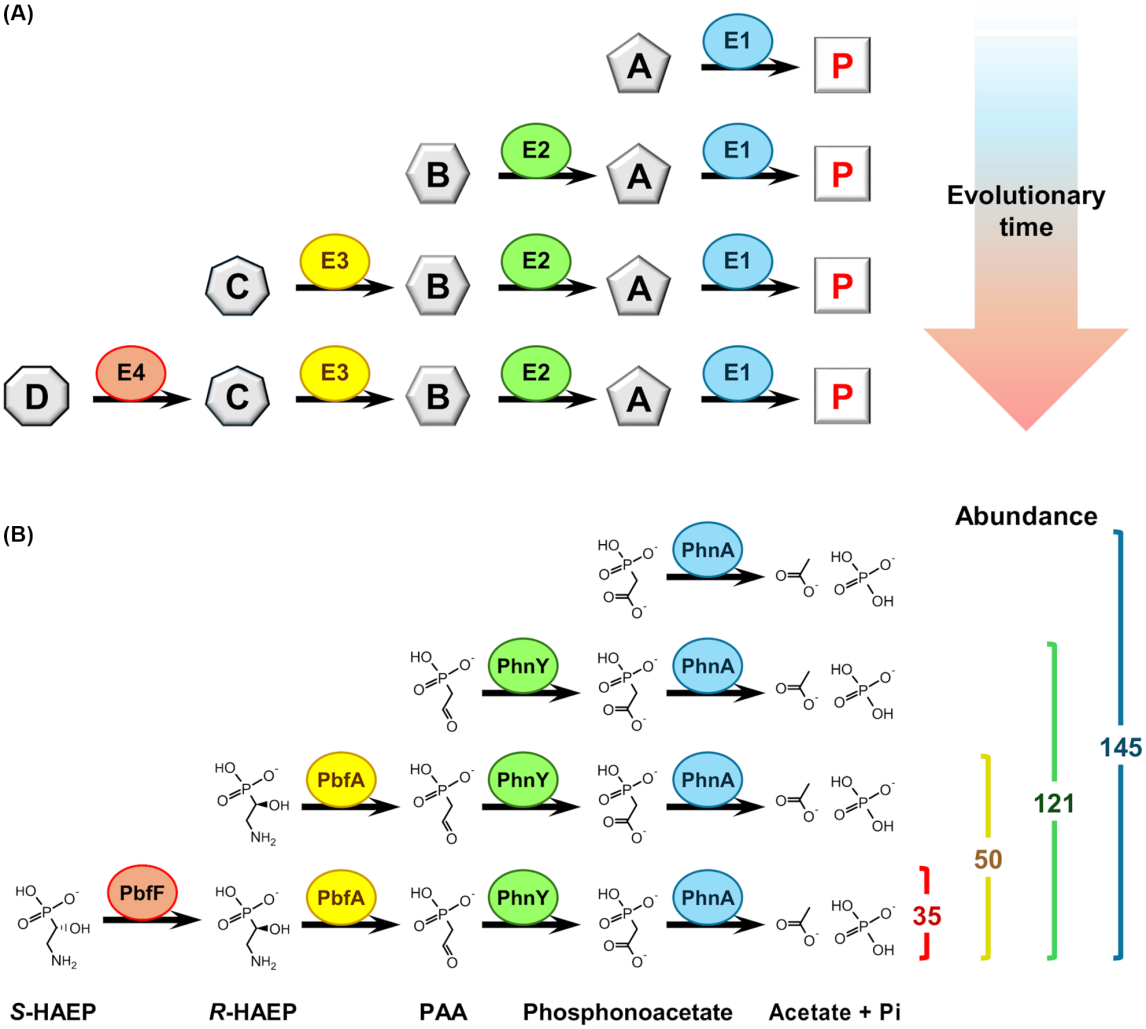
‘Retrograde’ emergence of the *S*‐HAEP degradation route. (A) The retrograde model of metabolic pathway evolution [60] posits that evolution of a pathway begins with a first enzyme (E1) able to transform an environmental compound A to yield a useful product P. The progressive depletion of A leads to a selective pressure for the development of a second enzyme (E2), which can obtain A from an available precursor B. When B becomes depleted, yet another enzyme (E3) can emerge, and so forth. (B) Development of the *S*‐HAEP degradation pathway. Numbers on the right refer to a census of *phnA* genes in 1098 complete genomes of *Hyphomicrobiales* and *Rhodobacterales*, annotated as “finished” in the IMG/M website.

The development of a route for the catabolism of *S*‐HAEP, inferable from the genomic distribution of the genes involved (*pbfF*, *pbfA*, *phnY*, *phnA*) is reminiscent of the so‐called “retrograde” mechanism of metabolic pathway evolution. The retrograde model was proposed by Horowitz [60] and posits that a pathway can build up backwards, one step at a time, exploiting chemical intermediates occurring in the environment (Fig. 10A). Evolution would begin with an enzyme able to perform a beneficial reaction using a substrate available in the environment. Upon depletion of this substrate, there would be evolutionary pressure for another enzyme to emerge, capable of replenishing the pools of substrate from some precursor, also environmentally available. Then there would be further pressure for the evolution of yet another enzyme to synthesize the precursor, and so on [60]. According to this model, the last enzyme in a linear pathway is the one that evolved first, whereas the first enzyme of the pathway evolved last (Fig. 10A).

Let us now consider the pathway leading from *S*‐HAEP to phosphate and acetate through the reactions of four enzymes (PbfF, PbfA, PhnY, PhnA). The last enzyme in the pathway (PhnA) may well have been the first enzyme to evolve. PhnA is useful for the degradation of phosphonoacetate (another presumed biogenic compound [61]) and homologs of *phnA* are widespread in a variety of bacteria [61, 62, 63]. Very often *phnA* is genomically clustered with *phnY*, whose gene product converts PAA (however formed) to phosphonoacetate. The *phnY‐phnA* association is presumably quite ancient, as it occurs in different bacterial phyla such as Proteobacteria, Terrabacteria, and Bacteroidetes. In comparison, the *pbfA‐phnY‐phnA* combination (required to degrade *R*‐HAEP) is only found in Proteobacteria (although *pbfA*, associated with *phnX*, also occurs in other phyla). Finally, PbfF seems to have emerged rather recently, as clusters that encode this enzyme, along with the previous three, are found only in two Alphaproteobacteria orders, the *Hyphomicrobiales* and *Rhodobacterales* (Table 1).

Remarkably, the relative abundance of the different cluster types within these two orders also seems consistent with a different degree of antiquity. In a set of 1098 complete genomes of *Hyphomicrobiales* and *Rhodobacterales*, we found a total of 145 close *phnA* homologs (Fig. 10B). In most instances (121/145) the *phnA* gene clustered with *phnY*. In 50 instances, the cluster also included *pbfA*. Finally, only 35 clusters containing all four genes were observed in this set of genomes (Fig. 10B).

## Concluding remarks

PbfF is a novel enzyme in phosphonate catabolism, serving to degrade *S*‐HAEP. PbfF is an unusual NAD‐dependent racemase; it is unrelated to other known isomerases and likely evolved from an ancestral d‐α‐hydroxyacid dehydrogenase. As in the case of PbfA (a lyase that apparently derived from a transaminase ancestor [21]), this shift in catalytic function would have been difficult to predict based solely on sequence criteria, but it was strongly suggested by the genomic context of the *pbfF* gene. The evolutionary origins of PbfF are presumably recent, as the enzyme is found only in two orders of *Alphaproteobacteria*. Overall, the emergence of a pathway for *S*‐HAEP‐degradation exemplifies the retrograde model of metabolic pathway evolution.

## Materials and methods

### Materials

Triethanolamine (TEA), Bis‐Tris Propane, and a bacterial NADP‐specific alcohol dehydrogenase (ADH; catalog # 49641) were purchased from Sigma‐Aldrich (now Merck; St. Louis, MO, USA). 2‐Aminoethylphosphonate (AEP) was from Wako chemicals (Osaka, Japan), NAD^+^ was from Roche (Basel, Switzerland), and NADH was from Alfa Aesar (Haverhill, MA, USA). The BIOMOL Green kit^®^ for phosphate detection was from Enzo Life Sciences (Farmingdale, NY, USA).

### Chemical synthesis of phosphonate compounds

Racemic HAEP was synthesized starting from vinylphosphonic acid, as described [64]; the (*S*)‐ and (*R*)‐ enantiomers of HAEP were prepared by methods reported in the same publication [64]. 2‐Hydroxyethylphosphonate (HEP) was also synthesized as described in the literature [65, 66].

### Bioinformatic analyses

As described in previous studies from our group [21, 22], gene context was explored using tools available at the Integrated Microbial Genomes & Microbiomes (IMG/M) website (https://img.jgi.doe.gov/m/) and AEP‐degradation clusters were primarily identified based on the colocalization of genes encoding the transaminase PhnW and either the hydrolase PhnX or the duo of enzymes PhnY/PhnA (see Fig. 1A). Typically, these clusters also contain genes coding for transporter proteins, transcriptional regulators, and occasionally ancillary enzymes such as PbfA and PbfD [21, 22]. This analysis led us to focus on a novel gene (which we termed *pbfF*) occurring in the clusters from several *Alphaproteobacteria* genomes.

The sequence of the PbfF protein was used for BLASTP analyses against the UniProt/Swissprot database to identify the closest homologous enzymes of known function. The PbfF sequences and those of their homologs were then aligned with ClustalX2. To estimate percent identities between couples of proteins, sequences were aligned with Needle (https://www.ebi.ac.uk/Tools/psa/emboss_needle/) and the number of identical positions in the alignment was divided by the length of the shorter sequence.

A census and classification of the *phnA*‐containing gene clusters in the *Hyphomicrobiales* and *Rhodobacterales* was obtained through IMG/MER (https://img.jgi.doe.gov/cgi‐bin/mer/main.cgi). As of May 2024, the database included a total of 1098 ‘finished’ genome sequences from the two orders. These genomes were searched by BLAST, using as a query the functionally validated PhnA from *Sinorhizobium meliloti* (GenBank: WP_010975822) [67], yielding 145 hits (111 in *Hyphomicrobiales* genomes and 34 in *Rhodobacterales* genomes). For each hit, the genome context was individually examined to detect the possible presence of *phnY*, *pbfA*, and *pbfF* homologs in the neighborhood (±10 genes) of the putative *phnA* gene.

### Expression and purification of recombinant proteins

Gene cloning, recombinant expression, and purification of the PhnX and PbfA enzymes from *Vibrio splendidus* have been described [21].

A gene encoding the PbfF enzyme from *Mesorhizobium plurifarium* (GenBank: CDX32929), codon‐optimized for expression in *Escherichia coli*, was synthesized and cloned into the NdeI/NotI restriction sites of a pET24‐C‐His‐tag vector by Proteogenix (Schiltigheim, France). The plasmid was then used to transform *E. coli* Tuner (DE3) cells for expression of the His‐tagged protein.

To this end, a preculture of transformed bacteria (10 mL) was grown overnight at 37 °C and then used to inoculate 1 L of Luria‐Bertani broth (LB) plus kanamycin (50 μg·mL^−1^), placed in a 5 L flask. The bacteria were grown at 37 °C until the OD_600_ reached 0.7–0.8. At that point, the temperature was lowered to 20 °C and protein expression was induced by adding 1 mm isopropyl‐β‐d‐1‐thiogalactopyranoside. The induced culture was grown for 20 h under vigorous shaking (148 rpm), after which the cells were harvested by centrifugation. Pellets were washed once in buffer A (50 mm Tris/HCl pH 7.9, 200 mm NaCl, 1 mm dithiothreitol, 10% glycerol) and then resuspended in the same buffer supplemented with 1 mm phenylmethylsulfonyl fluoride, 1 mm benzamidine, and 1 mg·mL^−1^ lysozyme. The cell suspension was first incubated for 30 min on ice and then lysed by freeze‐thawing, sonicated briefly to fragment DNA, and centrifuged for 40 min at 26 200 ***g*** (4 °C). Recombinant PbfF was then purified by Ni‐affinity chromatography. To this end, the supernatant was applied to a HisTrap™ Fast Flow 5 mL column (Cytiva, Marlborough, MA, USA) mounted on an AKTA Pure FPLC System (GE Healthcare, Stockholm, Sweden). Protein purity in the eluted fractions was assessed by SDS/PAGE, and fractions with purity >95% were pooled and dialyzed against buffer A. The PbfF concentration after dialysis was estimated based on an *ε*_280_ = 22 835 M^−1^·cm^−1^ calculated with ProtParam (http://web.expasy.org/protparam/); the final yield was 56 mg of purified protein per liter of bacterial culture. The protein was split into 1 mL‐aliquots and stored at −80 °C.

### Estimation of the NAD(P) content of recombinant PbfF

Recombinant PbfF did not show any significant absorbance at 340 nm, indicating the absence of bound NAD(P)H. To assess whether NAD(P)^+^ was bound to the purified protein, a 150‐μL aliquot of the PbfF stock (108 μm) was treated with NaBH_4_, and the spectra before and after the treatment were compared. Reduction of any protein‐bound NAD(P)^+^ was expected to increase the absorbance at 340 nm (the ability of NaBH_4_ to reduce NAD^+^ had been confirmed beforehand by treating a 100 μm solution of free NAD^+^). The observed increase in PbfF absorbance was minimal (≤0.005 OD), implying that the enzyme contained less than 0.008 moles of NAD(P)^+^ per mole of protein.

### Evaluating the PbfF oligomerization state by size‐exclusion chromatography (SEC)

The oligomerization state of purified PbfF was evaluated in an analytical SEC experiment. A 50‐μL aliquot of protein (108 μm, monomer concentration) was run through a Superdex^®^ 200 Increase 5/150 GL column (Cytiva) on an AKTA^®^ Pure System FPLC. A Tris–HCl buffer (50 mm Tris, 200 mm NaCl, pH 7.9) was used as the mobile phase, with a 0.3 mL·min^−1^ flow rate. A calibration chromatogram was recorded using a set of proteins of known molecular mass.

### Phosphate release assay

Phosphate release associated with enzymatic reactions was assessed using the BIOMOL Green^®^ kit (Enzo Life Sciences), according to the manufacturer's instructions.

For plate assays, various phosphonates were incubated in 50 μL of TEA‐HCl buffer (50 mm, pH 8.0), containing 5 mm MgCl_2_ and (when indicated) 1 μm PbfF, 1.6 μm PbfA, and/or 3 μm PhnX, as well as 0.3 mm NAD^+^ (or NADP^+^). The reactions were carried out at room temperature for one hour, after which, for each reaction mixture, 5 μL were added to 200 μL of BIOMOL Green^®^ reagent in the well of a 96‐well plate. Color development was assessed ~30 min later.

### Circular dichroism (CD) assessment of the HAEP racemization reaction

The PbfF‐catalyzed racemization of *S*‐ and *R*‐HAEP was probed by circular dichroism. Solutions of each of the two HAEP isomers (5 mm, pH adjusted to 8) were first prepared in a final volume of 3 mL. Subsequently, NAD^+^ (50 μm final) was added to each solution. The *S*‐HAEP solution was split into two aliquots (1.5 mL each), one of which was supplemented with 1 μm PbfF. The same was done with the *R*‐HAEP solution. All the samples (with and without the enzyme) were then incubated at room T for 75 min, after which they were ultrafiltered using a 2‐mL Amicon^®^ Ultra‐2 3 K Centricon device (Merck). Ultrafiltration of the aliquots containing PbfF allowed the removal of the enzyme, which would otherwise contribute substantially to the CD spectrum of the solution in the 200 nm region. The ultrafiltered solutions were finally placed into a quartz cuvette with a 0.5 cm path length and CD spectra were collected employing a thermostated J‐1500 CD spectrophotometer (Jasco Inc.). All the spectral measurements were corrected for water contribution.

### Kinetic assays of PbfF activity

The kinetics of the PbfF‐catalyzed conversion of *S*‐HAEP to *R*‐HAEP were monitored at pH 8.0 (25 °C), by coupling the isomerization with the sequential reactions of PbfA, PhnX, and of a NADP‐dependent alcohol dehydrogenase (ADH; EC 1.1.1.2). The choice of a NADP‐dependent ADH served to prevent interferences between the activity of PbfF and that of the dehydrogenase.

The coupling enzymes, PbfA (~18 μm) PhnX (~3.5 μm) and ADH (~6 U·mL^−1^), were used in large excess with respect to PbfF (typically 35 nm) to ensure that the overall reaction was limited by the racemization step. Control experiments, in which the concentration of PbfF varied between 17.5 and 70 nm, ensured that this was indeed the case. The reaction mixture also contained 50 mm TEA‐HCl (pH 8.0), 1 mm NAD^+^, ~0.2 mm NADPH, and 5 mm MgCl_2_, as well as *S*‐HAEP.

The reaction was typically initiated by adding PbfF last, after which the disappearance of NADPH was monitored at 340 nm. Kinetic data were analyzed by nonlinear least‐squares fitting to the appropriate kinetic equation using Sigma Plot (Systat Software Inc.).

### Structural prediction and docking analysis

We used AlphaFold 3 [39] to predict the 3D structure of PbfF both in the absence and in the presence of bound NAD^+^. The best models obtained in the two cases were in very good agreement (RMSD referred to α‐carbons = 1.294 Å).

We also applied P2Rank [40] to the model of NAD‐free PbfF to independently search for potential binding pockets in that structure. This analysis identified only one pocket, with a probability of 0.878, that encompassed entirely the NAD^+^ binding site predicted by AlphaFold 3. After these checks, the model of the PbfF monomer with bound NAD^+^ was used for comparisons with structurally related enzymes (see Results).

## Conflict of interest

The authors declare no conflict of interest.

## Author contributions

FR performed biochemical assays, kinetic measurements, and CD experiments. SC worked at the bioinformatic analyses, protein purification, and initial enzyme assays. GM performed the structural prediction and docking studies. TD chemically synthesized the HAEP isomers. JC contributed to data analysis. CR contributed to the design of experiments and to the analysis of biochemical data. AS supervised the CD measurements and the analysis of pertinent data. KP performed phosphonate synthesis and contributed to the experimental design. AP supervised the project, performed initial genome context analysis, and wrote the manuscript. All the authors have read and approved the final version of the paper.

## Peer review

The peer review history for this article is available at https://www.webofscience.com/api/gateway/wos/peer‐review/10.1111/febs.70130.

## Data Availability

The data that support the findings of this study are available in Figs 2, 3, 4, 5, 6, 7, 8, 9, 10 and Tables 1 and 2 of this article.
